# Predictive Role of Shoulder Imbalance and Residual C7PL–CSVL in Coronal Imbalance After Surgery for Severe and Rigid Scoliosis: A Retrospective Analysis

**DOI:** 10.1111/os.70269

**Published:** 2026-03-03

**Authors:** Honghao Yang, Haoshuang Geng, Jie Wang, Jixuan Huang, Yiqi Zhang, Yunsheng Wang, Yangpu Zhang, Lijin Zhou, Yong Hai

**Affiliations:** ^1^ Department of Orthopedic Surgery, Beijing Chao‐Yang Hospital Capital Medical University Beijing China

**Keywords:** coronal decompensation, coronal imbalance, predictors, risk factors, severe and rigid scoliosis, shoulder imbalance, trunk shift

## Abstract

**Objective:**

Coronal balance and shoulder balance affect the results of surgical treatments. Numerous studies on these topics in moderate scoliosis have been reported. However, the risk factors and the association between coronal imbalance and shoulder imbalance in complex spinal deformity remain unknown. The purpose of this study is to investigate whether shoulder imbalance as well as other radiographic factors could predict ultimate coronal imbalance in severe and rigid scoliosis.

**Methods:**

A retrospective study was conducted at our hospital between January 2009 and December 2018. Fifty‐one patients with severe and rigid thoracic/thoracolumbar scoliosis (main curve Cobb angle > 80° and flexibility < 25%) were recruited. Patients were divided into the Coronal balance (CB) group (C7PL‐CSVL ≤ 20 mm) and the Coronal imbalance (CIB) group (C7PL‐CSVL > 20 mm). Then, the patients in the CIB group were stratified based on the aggravation of C7PL‐CSVL for further subgroup analysis. Potential risk factors for coronal imbalance and decompensation, including shoulder height data and various radiographic parameters, were analyzed between groups and summarized in a quantitative predictive equation.

**Results:**

Of all patients, 43.1% (22/51) showed coronal imbalance at the last follow‐up. Univariate analysis showed that the following parameters were significantly greater in CIB group: shoulder height data including immediately postoperative radiographic shoulder height (RSH, *p* = 0.001), postoperative clavicle angle (CA, *p* = 0.000), and postoperative C7PL‐CSVL (*p* = 0.000). Logistic regression identified that immediately postoperative CA [odds ratio (OR) = 6.837, *p* = 0.008] and C7PL‐CSVL (OR = 1.071, *p* = 0.010) were the independent risk factors for ultimate coronal imbalance. The predictive equation was Risk Index = −5.277 + 1.922 × postoperative CA + 0.068 × postoperative C7PL‐CSVL, with positive and negative predictive values of 86.7% and 85.7%, respectively.

**Conclusions:**

The prevalence of coronal imbalance at the last follow‐up remained high in severe and rigid scoliosis. Postoperative shoulder imbalance and residual trunk shift could be used as predictors for ultimate coronal imbalance. The aggravation of coronal imbalance might represent a possible compensatory response to shoulder imbalance, as suggested by the observed adverse trends. However, this interpretation should be considered hypothetical, since a direct causal relationship could not be verified yet. Surgeons should pay attention to restore an appropriate relationship between curves in surgical planning for better results.

## Graphical abstract

The prevalence of coronal imbalance remained high in severe and rigid scoliosis. Postoperative shoulder imbalance and residual trunk shift could be used as predictors for ultimate coronal imbalance. The aggravation of coronal imbalance might be a compensation, trying to level the imbalanced shoulder and attain a truncal global balance.

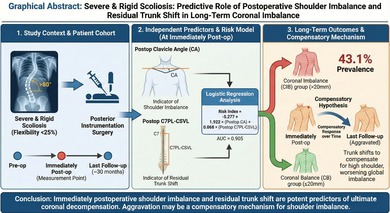

## 1 Background

Severe and rigid scoliosis is commonly defined as a main curve magnitude greater than 80° with flexibility less than 25%, and is often associated with rapid deformity progression and cardiopulmonary dysfunction [1]. Routine options for treatment include perioperative halo‐gravity traction, anterior release and posterior fusion with instrumentation, and spinal osteotomy, such as vertebral column resection (VCR) [2, 3, 4]. As the deformity in severe and rigid scoliosis is usually too stiff and hard to correct, radical release or multiple osteotomies of the spinal column are needed. Also, surgeons have placed too much emphasis on the correction of the frontal curve while the restoration of global coronal balance is ignored.

Coronal imbalance or decompensation, a complication of scoliosis correction, is defined as the shift of the trunk in the frontal plane [5]. If it is not properly addressed in surgery, coronal imbalance may result in dissatisfaction with treatments, inferior self‐assessment, and the requirement for revision surgery in later years [6]. Therefore, attention should be paid to the causation and mechanism of coronal imbalance in order to prevent this complication.

Numerous risk factor analyses have been reported that the overcorrection of thoracic curve, the flexibility of lumbar curve, and the selection of fusion levels were related to the coronal imbalance [7, 8, 9]. However, these conclusions were primarily drawn from studies on moderate and flexible scoliosis (Cobb angle < 80°). Whether these findings remain applicable to severe and rigid scoliosis remains unclear. In this subgroup, the combination of a large curve magnitude and poor flexibility leads to limited compensatory potential of unfused segments, which profoundly alters the postoperative remodeling process and the restoration of global balance [10]. Consequently, these patients might be more susceptible to aggravating the trunk shift and increasing the prevalence of coronal imbalance after surgery.

Furthermore, there is a lack of data that focuses on the impact of shoulder imbalance on changes of trunk alignment. Previous studies have reported that the incidence of postoperative shoulder imbalance in severe and rigid scoliosis was significantly higher than moderate scoliosis (32.65% vs. 8.93%), and the subsequent change of coronal alignment was speculated as a compensatory mechanism associated with shoulder imbalance [1, 11, 12]. Cao et al. and Matsumoto et al. additionally reported that postoperative shoulder imbalance could be accommodated by the development of distal adding‐on or trunk shift [13, 14]. Yet, these mechanisms may behave differently in severe and rigid spine deformities, where compensatory adjustments are greatly restricted. Hence, a comprehensive analysis focusing specifically on severe and rigid scoliosis is both necessary and clinically meaningful.

The present study aimed to evaluate the relationship between shoulder imbalance and postoperative coronal imbalance in patients with severe and rigid scoliosis, and to identify independent radiographic predictors for ultimate coronal imbalance. Furthermore, this study sought to establish a quantitative predictive equation, providing surgeons with a practical tool to anticipate postoperative coronal imbalance and to optimize surgical planning in this challenging population.

## 2 Methods

### 2.1 Patient Cohort

A retrospective study was conducted at our hospital between January 2009 and December 2018 to identify patients with severe and rigid scoliosis. The inclusion criteria were patients who underwent posterior instrumentation with a minimum 24‐month follow‐up, with a major curve magnitude greater than 80° and a flexibility of less than 25%. Patients with a previous history of spinal surgery were excluded. Demographic information including age, sex, and etiology was collected.

### 2.2 Radiographic and Clinical Assessment

Full‐length standing posteroanterior and lateral radiographs of the spine were obtained preoperatively, immediately postoperatively, and at the last follow‐up, along with preoperative side‐bending radiographs for radiographic assessment. All radiographs were obtained in a standardized standing position, with patients looking straight ahead and both upper limbs resting naturally. Two independent spine surgeons performed all radiographic measurements, and the mean values were used for statistical analysis. The radiographic parameters relevant to shoulder height were radiographic shoulder height (RSH) and clavicle angle (CA). RSH indicated the height difference in the bilateral soft tissue shadows directly superior to the acromioclavicular joint [15]. Positive values for RSH represented the left shoulder up. Clavicle angle indicated the angle between the horizontal line and the tangential line connecting the highest points of each clavicle [15]. Positive values for CA represented the left clavicle up. Shoulder imbalance was defined as the absolute value of RSH greater than 20 mm or CA greater than 2.5° [16, 17]. Other radiographic and surgical variables were collected, including the Cobb angle of proximal thoracic curve (PTC), main thoracic curve (MTC), and thoracolumbar/lumbar curve (TL/LC), the ratio of MTC to TL/LC, apical vertebral translation (AVT) of MTC, apical vertebral rotation (AVR) of MTC using the Nash‐Moe method [18], upper end vertebra (UEV)‐upper instrumented vertebra (UIV) distance, lower end vertebra (LEV)‐lowest instrumented vertebra (LIV) distance, traction, osteotomy type, UIV/LIV criteria, bracing, and the correction rate of PTC, MTC as well as TL/LC. The flexibility of the curves was assessed by side‐bending radiographs.

C7PL‐CSVL was defined as the distance from the C7 plumb line (C7PL) to the center sacral vertical line (CSVL). Coronal imbalance was defined as the absolute value of C7PL‐CSVL greater than 20 mm. Positive values for C7PL‐CSVL represented the right‐sided trunk shift, et vice versa. Based on the C7PL‐CSVL at the last follow‐up, patients were divided into two groups: Coronal Balance (CB) group (C7PL‐CSVL ≤ 20 mm) and Coronal Imbalance (CIB) group (C7PL‐CSVL > 20 mm). Additionally, a subgroup analysis was performed for patients in the CIB group. According to Hwang et al. [19], patients were stratified based on the change in C7PL–CSVL between the initial and the last visits: the Aggravated group (aggravation > 20 mm) and the Non‐aggravated group (aggravation ≤ 20 mm).

## 3 Statistical Analysis

All statistical analyses were performed using SPSS version 25.0 (Chicago, IL, USA). According to previous studies, positive absolute values were assigned for statistical analysis of all variables [20, 21, 22, 23]. Inter‐rater reliability of RSH and CA was reported using the intraclass correlation coefficient (ICC) with 95% CIs. Distributions of variables were presented as mean and standard deviation. Repeated measures ANOVA was used to assess the changes in radiographic parameters over time. Independent Student *t*‐test was utilized to analyze continuous data between groups. Fisher's exact test was applied to analyze categorical variables. Factors with *p* < 0.05 were considered statistically significant. Subsequent factor analysis was performed by including potential factors in the binary logistic regression model to identify independent risk factors for coronal imbalance. Furthermore, a predictive equation was developed based on the results of the logistic regression. The receiver operating characteristic (ROC) curve was created to determine the cutoff value of the equation. Calibration was assessed using the Hosmer‐Lemeshow goodness of fit test and the calibration plot.

## 4 Results

### 4.1 Baseline Characteristics

A total of 51 patients (36 female, 15 male) with severe and rigid thoracic or thoracolumbar scoliosis were recruited (Table 1). The mean age at surgery was 21.63 ± 5.30 years (range 13–31 years). The mean follow‐up was 30.57 ± 8.95 months (range 24–48 months). Of the scoliosis cases, 30 were idiopathic, and 21 were congenital without lumbosacral hemivertebra. The main curve Cobb angle was 112.80° ± 20.55° (range, 80.20°–142.40°), with average flexibility of 13.9%. There were no significant differences in age, sex, etiology, fusion levels, and the location of LIV between the CB and CIB group (*p* > 0.05). The comparison of key factors in surgical details between CB group and CIB group was demonstrated in Table 2, which showed that there were no significant differences in traction (*p* = 0.850), osteotomy type (*p* = 0.925), UIV criteria (*p* = 0.817), LIV criteria (*p* = 0.065), and bracing (*p* = 1.000). Also, there were no significant differences in baseline characteristics between the Aggravated group and the non‐Aggravated group.

**TABLE 1:** Demographic data and scoliotic curve parameters for the patient cohort.

Parameters	Mean ± SD/*n* (%)
Age (years)	21.63 ± 5.30
Sex	
Male	15 (29.4%)
Female	36 (70.6%)
Etiology	
Idiopathic	30 (58.8%)
Congenital	21 (41.2%)
Cobb angle (°)	112.80 ± 20.55
Flexibility	13.9%
Fusion levels	13.12 ± 2.03
Location of lowest instrumented vertebra	
L2	1 (2.0%)
L3	9 (17.6%)
L4	26 (51.0%)
L5	14 (27.4%)
S1	1 (2.0%)

**TABLE 2:** Comparison of key factors in surgical details between CB group and CIB group.

	CB group (*n* = 29)		CIB group (*n* = 22)		*p*
Traction					0.850
Halo‐gravity	19	(65.5%)	16	(72.7%)	
Halo‐pelvic	2	(6.9%)	1	(4.5%)	
Osteotomy type					0.925
Ponte	11	(37.9%)	8	(36.4%)	
Pedicle subtraction osteotomy	2	(6.9%)	1	(4.5%)	
Vertebral column resection	16	(55.2%)	13	(59.1%)	
UIV criteria					0.817
UEV	5	(17.2%)	4	(18.2%)	
UEV + 1	11	(37.9%)	6	(27.3%)	
UEV + 2	8	(27.6%)	8	(36.4%)	
UEV + 3	4	(13.8%)	3	(13.6%)	
UEV + 4	1	(3.4%)	1	(4.5%)	
UEV‐LIV (segments)	1.48 ± 1.09		1.59 ± 1.10		0.725
LIV criteria					0.065
LEV +1	0	(0.0%)	2	(9.1%)	
LEV	3	(10.3%)	6	(27.3%)	
LEV‐1	15	(51.7%)	5	(22.7%)	
LEV‐2	9	(31.0%)	5	(22.7%)	
LEV‐3	2	(6.9%)	2	(9.1%)	
LEV‐4	0	(0.0%)	2	(9.1%)	
LEV‐LIV (segments)	1.34 ± 0.77		1.23 ± 1.45		0.832
Postoperative bracing	29	(100.0%)	22	(100.0%)	1.000

Abbreviations: LEV, lower end vertebra; LIV, lowest instrumented vertebra; UEV, upper end vertebra; UIV, upper instrumented vertebra.

### 4.2 CB Group Versus CIB Group

Coronal imbalance at the last follow‐up was detected in 22 patients (43.1%). Sixteen of the 22 patients (72.7%) presented immediately postoperative coronal imbalance, whereas no sufficient spontaneous compensation was observed at the last follow‐up, and the other six patients (27.3%) developed new coronal imbalance during the follow‐up period.

**FIGURE 1:**
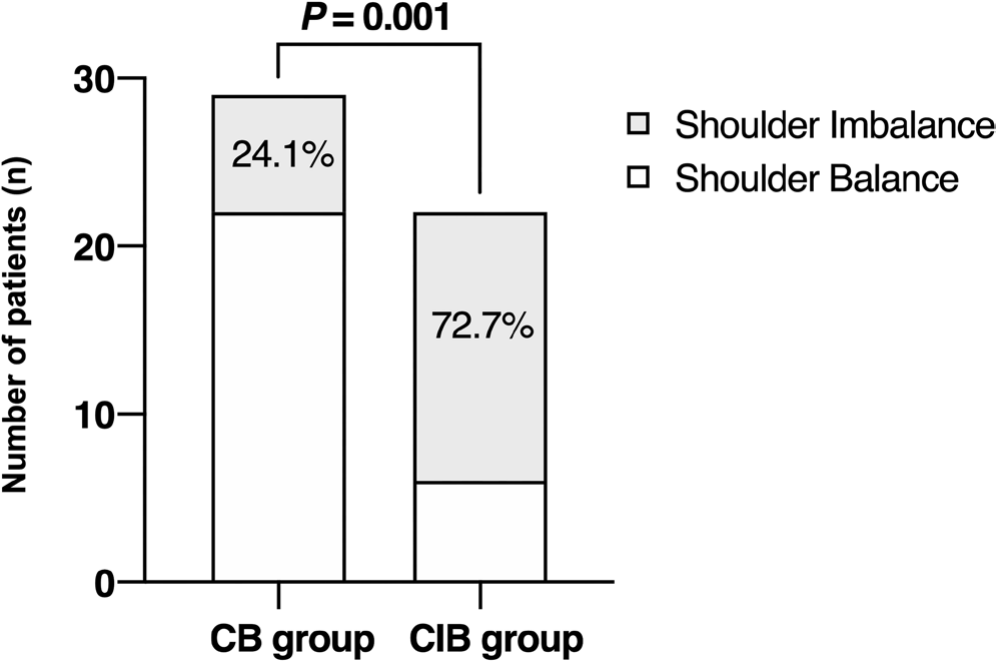
Comparison of the incidence of postoperative shoulder imbalance between CB group and CIB group.

**FIGURE 2:**
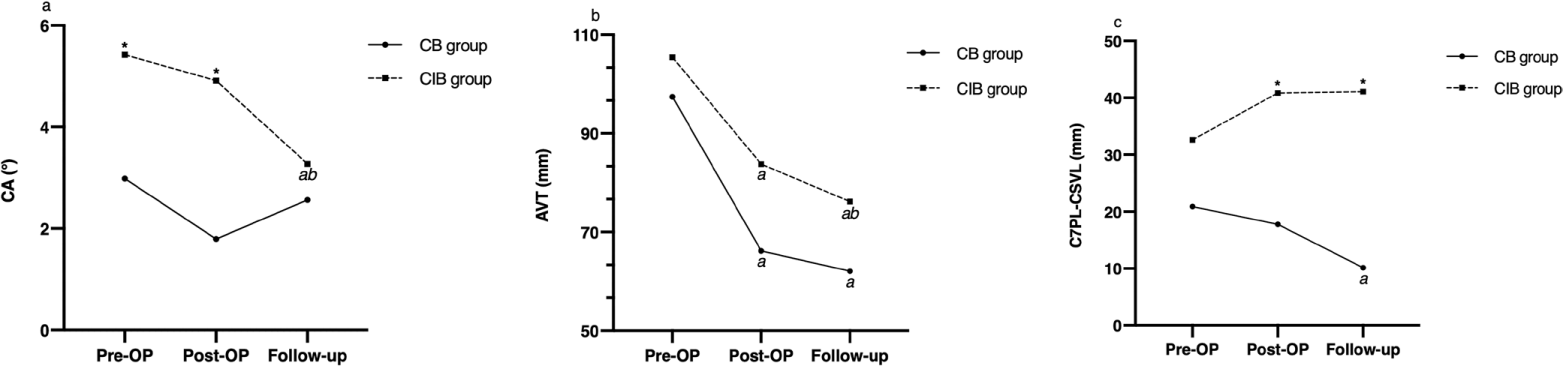
The changes of radiographic parameters over time in CB group and CIB group: (a) clavicle angle, CA; (b) apical vertebral translation, AVT; (c) C7PL‐CSVL. *Statistically significant between two groups. *a* Significantly different from the Pre‐OP value; *b* Significantly different from the Post‐OP value.

The incidence of postoperative shoulder imbalance was significantly higher in the CIB group compared with the CB group (72.7% vs. 24.1%, *p* = 0.001) (Figure 1). To further evaluate the role of shoulder height parameters in predicting coronal imbalance, univariate analyses were performed, and the results are presented in Figure 2 and Table 3. In the CB group, RSH and CA remained relatively stable around their preoperative levels. In contrast, the CIB group demonstrated a significant decline in CA from the initial visit to the last follow‐up (*p* = 0.028), with a similar downward trend observed in RSH, although it did not reach statistical significance (*p* > 0.05). Also, patients in the CIB group showed significantly spontaneous compensation for AVT during the follow‐up (from 83.79 ± 34.39 to 76.16 ± 36.83, *p* = 0.02), but this phenomenon was not remarkable in the CB group (*p* > 0.05). However, the considerable postoperative C7PL–CSVL in the CIB group showed no significant improvement during follow‐up (−0.26 ± 3.67 mm, *p* > 0.05). Significant differences between groups were identified for the following parameters: The shoulder height data including immediately postoperative RSH (10.26 ± 8.02 mm vs. 22.26 ± 13.89 mm, *p* = 0.001) and postoperative CA (1.79° ± 1.50° vs. 4.91° ± 2.88°, *p* = 0.000), preoperative ratio of MTC to TL/LC (2.59 ± 1.00 vs. 4.00 ± 2.63, *p* = 0.025) and postoperative C7PL‐CSVL (17.79 ± 13.02 mm vs. 40.85 ± 24.28 mm, *p* = 0.000). The inter‐rater reliability of RSH and CA measurement was 0.903 (0.872–0.929) and 0.911 (0.883–0.935), respectively.

**TABLE 3:** Comparison of radiographic data between CB group and CIB group.

	CB group (*n* = 29)	CIB group (*n* = 22)	
Shoulder height parameters			*p*
RSH (mm)			
Pre‐OP	15.35 ± 8.60	26.16 ± 22.62	**0.043**
Post‐OP	10.26 ± 8.02	22.26 ± 13.89	**0.001**
Follow‐up	11.15 ± 11.42	17.92 ± 10.36	**0.034**
CA (°)			
Pre‐OP	2.98 ± 1.93	5.42 ± 5.35	**0.027**
Post‐OP	1.79 ± 1.50	4.91 ± 2.88	**0.000**
Follow‐up	2.56 ± 2.80	3.27 ± 2.41^ab^	0.344
General parameters			
PTC (°)			
Pre‐OP	48.81 ± 21.91	47.51 ± 25.67	0.846
Post‐OP	31.37 ± 17.48^a^	32.35 ± 19.40^a^	0.850
Follow‐up	31.97 ± 16.54^a^	29.7 ± 21.00^a^	0.670
MTC (°)			
Pre‐OP	111.32 ± 21.50	114.75 ± 19.54	0.559
Post‐OP	53.72 ± 29.81^a^	59.66 ± 19.61^a^	0.396
Follow‐up	52.34 ± 27.44^a^	58.26 ± 24.15^a^	0.426
AVT (mm)			
Pre‐OP	97.40 ± 29.14	105.41 ± 32.20	0.358
Post‐OP	66.15 ± 31.35^a^	83.79 ± 34.39^a^	0.062
Follow‐up	62.09 ± 28.11^a^	76.16 ± 36.83^ab^	0.128
AVR	3.31 ± 0.60	3.32 ± 0.78	0.968
TL/LC (°)			
Pre‐OP	48.02 ± 16.62	37.44 ± 20.43	0.054
Post‐OP	19.19 ± 15.55^a^	19.08 ± 8.17^a^	0.137
Follow‐up	18.47 ± 12.93^a^	15.69 ± 8.26^a^	0.355
Ratio of MTC to TL/LC			
Pre‐OP	2.59 ± 1.00	4.00 ± 2.63	**0.025**
Post‐OP	3.86 ± 2.12	4.93 ± 3.36	0.198
Follow‐up	3.66 ± 2.07	5.12 ± 3.72	0.109
C7PL‐CSVL (mm)			
Pre‐OP	20.90 ± 17.92	32.59 ± 22.36	0.051
Post‐OP	17.79 ± 13.02	40.85 ± 24.28	**0.000**
Follow‐up	10.13 ± 6.63 ^a^	41.11 ± 21.56	**0.000**
Flexibility of PTC	28.5%	26.5%	0.451
Flexibility of MTC	12.3%	14.1%	0.465
Flexibility of TL/LC	41.3%	39.5%	0.355
Correction rate of PTC	33.2%	37.3%	0.563
Correction rate of MTC	54.2%	50.1%	0.443
Correction rate of TL/LC	63.1%	47.9%	0.077

*Note:* Bold values indicate statistically significance in *t*‐test (*p* < 0.05). ^a^Significantly different from the Pre‐OP value; ^b^Significantly different from the Post‐OP value (*p* < 0.05).

Abbreviations: AVR, apical vertebral rotation; AVT, apical vertebral translation; CA, clavicle angle; LEV, lower end vertebra; LIV, lowest instrumented vertebra; MTC, main thoracic curve; Post‐OP, postoperative; Pre‐OP, preoperative; PTC, proximal thoracic curve; RSH, radiographic shoulder height; TL/LC, thoracolumbar/lumbar curve.

**FIGURE 3:**
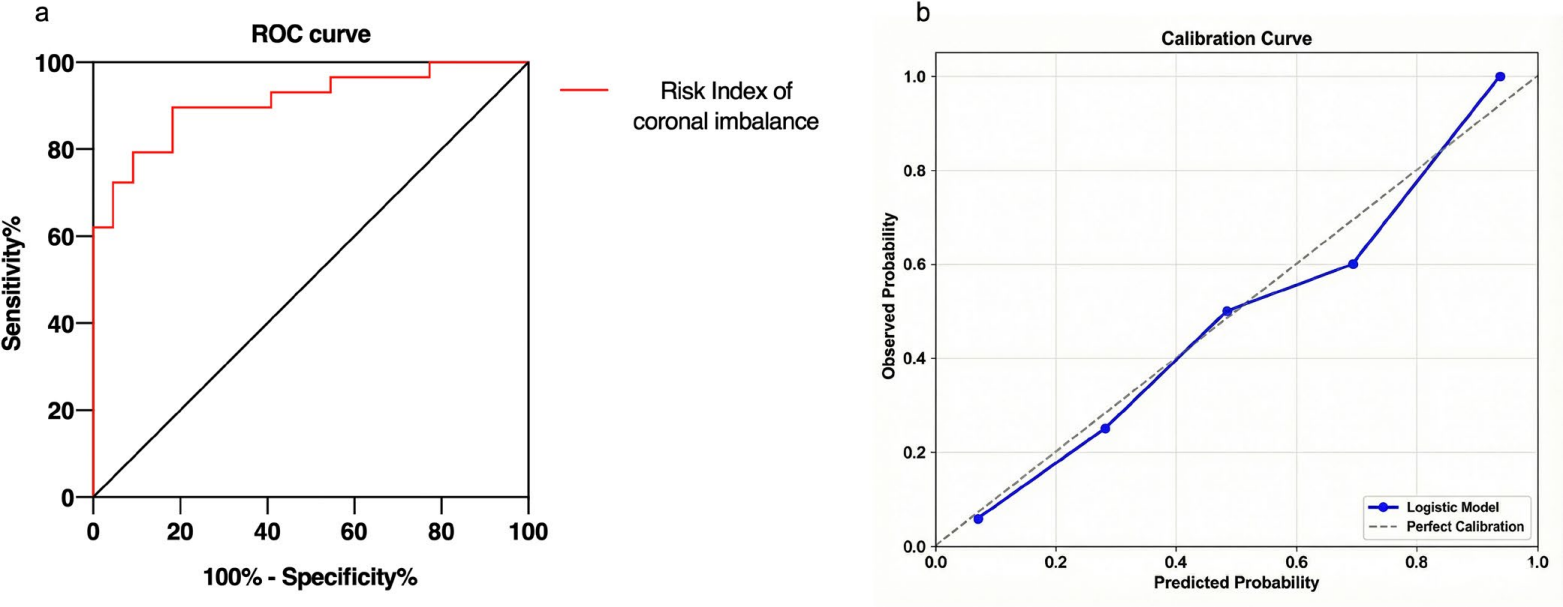
The predictive performance of the logistic regression model. (a) Receiver operating characteristic curve of the Risk Index for predicting ultimate coronal imbalance. The area under the curve was 0.905, with an optimal cutoff value of 3.15. (b) Calibration plot comparing predicted probabilities with observed outcomes. The dashed diagonal line represents perfect calibration. The model demonstrated good calibration, indicated by a non‐significant Hosmer‐Lemeshow test (*p* = 0.844).

**FIGURE 4:**
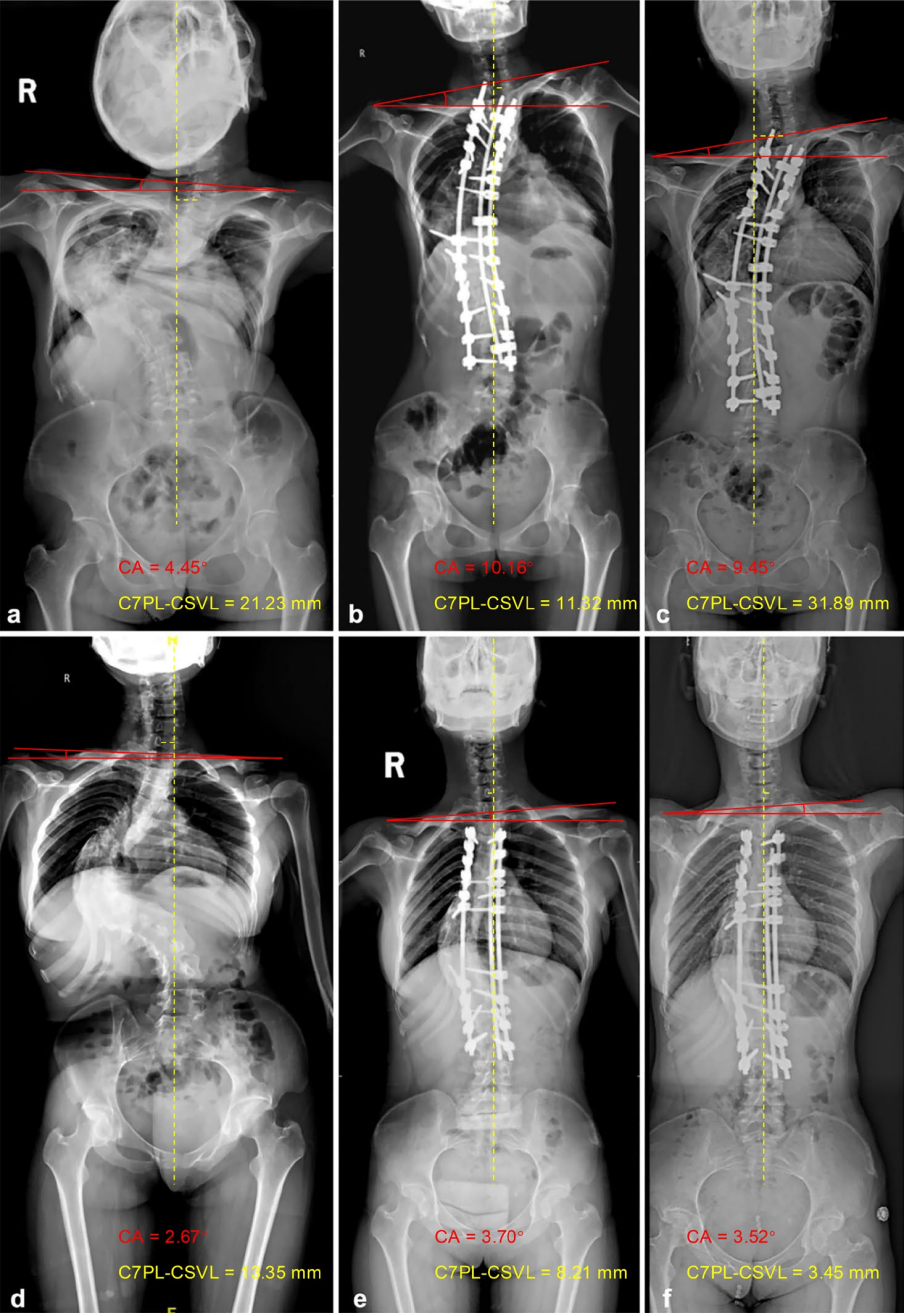
(a–c) The preoperative, immediately postoperative, and the last follow‐up radiographs of a 38‐year‐old female who was diagnosed with severe and rigid idiopathic scoliosis and underwent posterior instrumentation. The postoperative CA was 10.16° and the postoperative C7PL‐CSVL was 11.32 mm. Based on the predictive equation, the Risk Index of coronal imbalance was 15.02 (−5.277 + 1.922 × 10.16 + 0.068 × 11.32), which was greater than the cutoff value (3.15), indicating a high risk of coronal imbalance during the follow‐up. At the last follow‐up, coronal imbalance was observed with C7PL‐CSVL of 31.89 mm; (d–f) The preoperative, immediately postoperative, and the last follow‐up radiographs of a 26‐year‐old female who was diagnosed with severe and rigid congenital scoliosis and underwent posterior instrumentation. The postoperative CA was 3.70° and the postoperative C7PL‐CSVL was 8.21 mm. Based on the predictive equation, the Risk Index of coronal imbalance was 2.39 (−5.277 + 1.922 × 3.70 + 0.068 × 8.21), which was less than the cutoff value (3.15), indicating a low risk of coronal imbalance during the follow‐up. At the last follow‐up, coronal balance was maintained well with C7PL‐CSVL of 3.45 mm.

The results of multivariate binary logistic regression revealed that postoperative CA (OR = 6.837, 95% CI: 1.659–28.187, *p* = 0.008) and postoperative C7PL‐CSVL (OR = 1.071, 95% CI: 1.016–1.128, *p* = 0.01) were independent risk factors for coronal imbalance at the last follow‐up (Table 4). Based on the logistic regression analysis, a predictive equation was established to estimate the occurrence of ultimate coronal imbalance. The full logistic equation for Risk Index of coronal imbalance was defined as −5.277 + 1.922 × postoperative CA + 0.068 × postoperative C7PL‐CSVL. The ROC curve was illustrated in Figure 3a, and the area under curve (AUC) was 0.905 (95% CI: 0.818–0.978, *p* < 0.001). The optimal cutoff value of the Risk Index was 3.15, with sensitivity of 81.8% and specificity of 89.7%. The positive and negative predictive values were 86.7% and 85.7%, respectively. The model demonstrated good calibration with a Hosmer‐Lemeshow P‐value of 0.844. The Brier score was 0.131, reflecting good overall performance. The calibration plot (Figure 3b) showed that the predicted probabilities were closely aligned with the observed outcome frequencies. Example cases were presented in Figure 4.

**TABLE 4:** Independent risk factors for coronal imbalance at the final follow‐up.

Variables	B	SE	Wald	*p*	Odds ratio	95% CI	
						Lower limit	Upper limit
Postoperative CA	1.922	0.723	7.076	0.008	6.837	1.659	28.187
Postoperative C7PL‐CSVL	0.068	0.027	6.585	0.010	1.071	1.016	1.128
Constant	−5.277	1.567	11.34	0.001	0.005		

Abbreviation: CA, clavicle angle.

### 4.3 Aggravated Group Versus Non‐Aggravated Group

The incidence of coronal decompensation was 50.0% (11/22). Seven of the eleven patients (63.6%) had no preoperative coronal imbalance, and this decompensation might be iatrogenic.

The results of the univariate analysis were summarized in Table 5. During the follow‐up, greater spontaneous compensation for PTC (5.60° vs. 0.32°) and AVT (10.89 mm vs. 4.36 mm) was detected in non‐Aggravated group than Aggravated group, although it was not statistically significant (*p* > 0.05). Significant differences between Aggravated group and non‐Aggravated group were postoperative CA (6.13° ± 2.90° vs. 3.70° ± 2.41°, *p* = 0.045) and preoperative C7PL‐CSVL (20.94 ± 20.48 mm vs. 44.25 ± 18.20 mm, *p* = 0.011).

**TABLE 5:** Comparison of radiographic data between non‐Aggravated group and Aggravated group.

	non‐Aggravated group (*n* = 11)	Aggravated group (*n* = 11)	
Shoulder height parameters			*p*
RSH (mm)			
Pre‐OP	24.72 ± 19.86	27.59 ± 25.98	0.774
Post‐OP	17.58 ± 11.24	26.93 ± 15.20	0.117
Follow‐up	16.93 ± 9.22	18.91 ± 11.75	0.665
CA (°)			
Pre‐OP	4.90 ± 4.27	5.93 ± 6.43	0.663
Post‐OP	3.70 ± 2.41	6.13 ± 2.90	**0.045**
Follow‐up	3.09 ± 1.96	3.46 ± 2.88	0.729
General parameters			
PTC (°)			
Pre‐OP	45.75 ± 21.38	49.27 ± 30.33	0.756
Post‐OP	32.46 ± 20.77^a^	32.25 ± 18.94^a^	0.980
Follow‐up	26.86 ± 20.91^a^	32.56 ± 21.70^a^	0.538
MTC (°)			
Pre‐OP	111.35 ± 18.65	118.16 ± 20.69	0.426
Post‐OP	58.05 ± 23.15^a^	61.27 ± 16.31^a^	0.710
Follow‐up	54.87 ± 28.72^a^	61.65 ± 19.35^a^	0.523
AVT (mm)			
Pre‐OP	105.28 ± 37.95	105.54 ± 27.15	0.985
Post‐OP	79.64 ± 38.45^a^	87.93 ± 31.11^a^	0.585
Follow‐up	68.76 ± 38.88^a^	83.57 ± 34.88^a^	0.358
AVR	3.36 ± 0.81	3.27 ± 0.79	0.792
TL/LC (°)			
Pre‐OP	38.16 ± 23.46	36.71 ± 18.04	0.872
Post‐OP	12.72 ± 7.88^a^	15.45 ± 8.61^a^	0.447
Follow‐up	15.47 ± 7.38^a^	15.90 ± 9.41^a^	0.907
Ratio of MTC to TL/LC			
Pre‐OP	3.83 ± 2.92	4.17 ± 2.43	0.766
Post‐OP	5.51 ± 4.28	4.35 ± 2.17	0.433
Follow‐up	4.16 ± 2.66	6.08 ± 4.47	0.237
C7PL‐CSVL (mm)			
Pre‐OP	44.25 ± 18.20	20.94 ± 20.48	**0.011**
Post‐OP	42.53 ± 24.40	39.17 ± 25.24^a^	0.754
Follow‐up	32.80 ± 17.42^a^	49.42 ± 22.82^a^	0.069
LEV‐LIV (segments)	1.27 ± 1.85	1.18 ± 0.98	0.887
Flexibility of PTC	26.3%	28.2%	0.435
Flexibility of MTC	16.6%	18.3%	0.399
Flexibility of TL/LC	41.2%	38.1%	0.645
Correction rate of PTC	44.9%	29.6%	0.255
Correction rate of MTC	52.6%	47.6%	0.525
Correction rate of TL/LC	46.2%	50.6%	0.634

*Note:* Bold values indicate statistically significance in *t*‐test (*p* < 0.05). ^a^significantly different from the Pre‐OP value; ^b^Significantly different from the Post‐OP value (*p* < 0.05).

Abbreviations: AVR, apical vertebral rotation; AVT, apical vertebral translation; CA, clavicle angle; LEV, lower end vertebra; LIV, lowest instrumented vertebra; MTC, main thoracic curve; Post‐OP, postoperative; Pre‐OP, preoperative; PTC, proximal thoracic curve; RSH, radiographic shoulder height; TL/LC, thoracolumbar/lumbar curve.

Further multivariate binary logistic regression confirmed that the independent risk factor for coronal decompensation was postoperative CA > 4.84° (B = 2.622, OR 13.77, 95% CI 1.286–147.335, *p* = 0.03).

## 5 Discussion

### 5.1 Clinical Context

Trunk balance, which influences the self‐image and life quality of patients with scoliosis, is a vital evaluation indicator for surgical effects [24]. Coronal imbalance frequently occurred or aggravated after correction and instrumentation of severe scoliosis. Among patients with moderate scoliosis, the incidence of this complication ranged from 28.0% to 46.3% in the early postoperative period but decreased to 2.0%–13.8% at the last follow‐up [25, 26, 27, 28]. However, we found that the prevalence of coronal imbalance remained high (43.1%) at the last follow‐up in this study. The discrepancies between our study and previous researches may be attributed to the difference in study subjects. Due to the severe and stiff scoliotic curve, the inadequate compensatory capability failed to improve their trunk balance spontaneously during the follow‐up period. Poor spinal alignment could lead to back pain, further decompensation, and functional disability that requires revision surgery [19, 26, 29]. In order to obtain a satisfactory clinical outcome, it is crucial to investigate the predictors for the onset and aggravation of coronal imbalance in patients with severe and rigid scoliosis.

Numerous studies have reported various causative factors for coronal imbalance, including overcorrection of thoracic curve, a low MTC to TL/LC ratio, and inappropriate selection of LIV [30, 31, 32]. However, most studies were performed solely in patients with moderate and flexible scoliotic curves. It remained unclear whether previous conclusions are applicable to patients with complex spinal deformity. In the present study, none of these parameters showed a significant correlation with coronal imbalance. Therefore, historical data were analyzed to identify other potential factors associated with ultimate coronal imbalance or decompensation in patients with severe and rigid scoliosis.

### 5.2 Adverse Trends Between Shoulder Imbalance and Trunk Shift

The association between shoulder imbalance and the change of coronal alignment was rarely concerned [11, 13]. The compensation for shoulder imbalance by spontaneous PTC correction, loss of MTC correction, and deterioration of adding‐on or trunk shift was observed by Koller et al. [12]. Consistently, we found that the incidence of postoperative shoulder imbalance was significantly different between patients with or without coronal imbalance at the follow‐up (*p* = 0.02), and previous causative analyses involving shoulder height parameters were scarce; this was the first study that shed light on the shoulder height parameters in predicting coronal imbalance and decompensation in patients with severe and rigid scoliosis.

In the current study, between CB group and CIB group, the shoulder height parameters, immediately postoperative RSH and CA, were significantly greater in patients with coronal imbalance, which was consistent with the results of Chen et al. [33]. It suggested that postoperative shoulder imbalance might play a critical role in affecting the subsequent trunk alignment during the follow‐up. Further logistic regression analysis confirmed that the postoperative CA was an independent risk factor for the presence of coronal imbalance. Also, we noticed that 68.1% (15/22) of patients' C7PL shifted towards the side of the higher shoulder. Accordingly, the presence of large C7PL‐CSVL might be the consequence of trunk shift, trying to level the imbalanced shoulder and attain a truncal global balance, which was following the hypothesis of Trobisch et al. [11].

**FIGURE 5:**
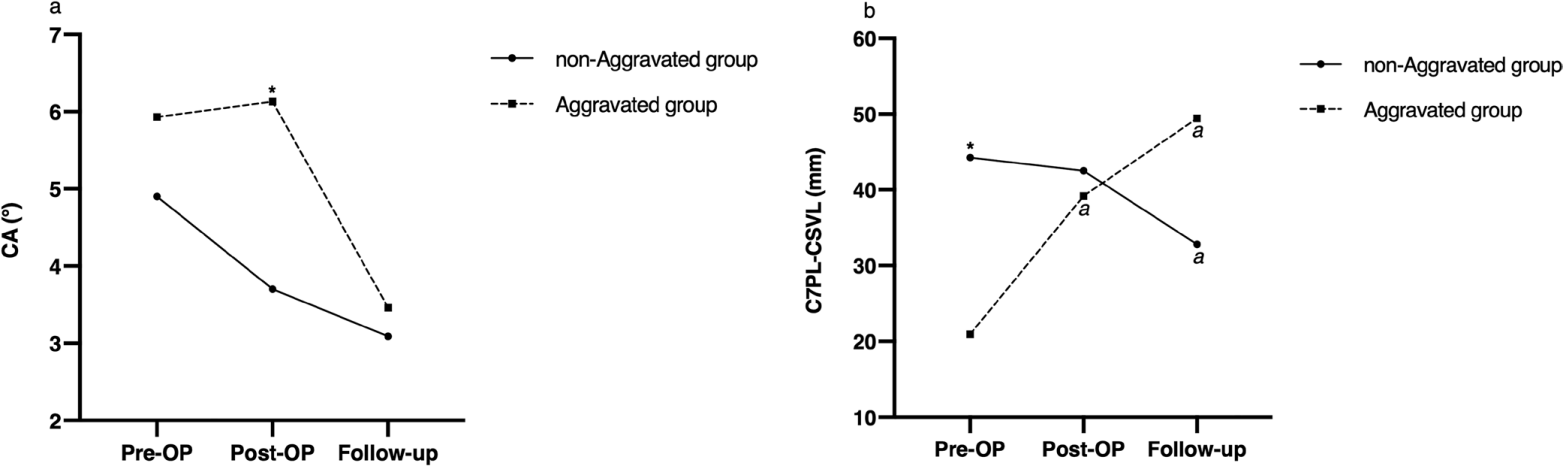
The changes of radiographic parameters over time in Aggravated group and non‐Aggravated group: (a) clavicle angle, CA; (b) C7PL‐CSVL. *Statistically significant between two groups. Significantly different from the Pre‐OP value.

**FIGURE 6:**
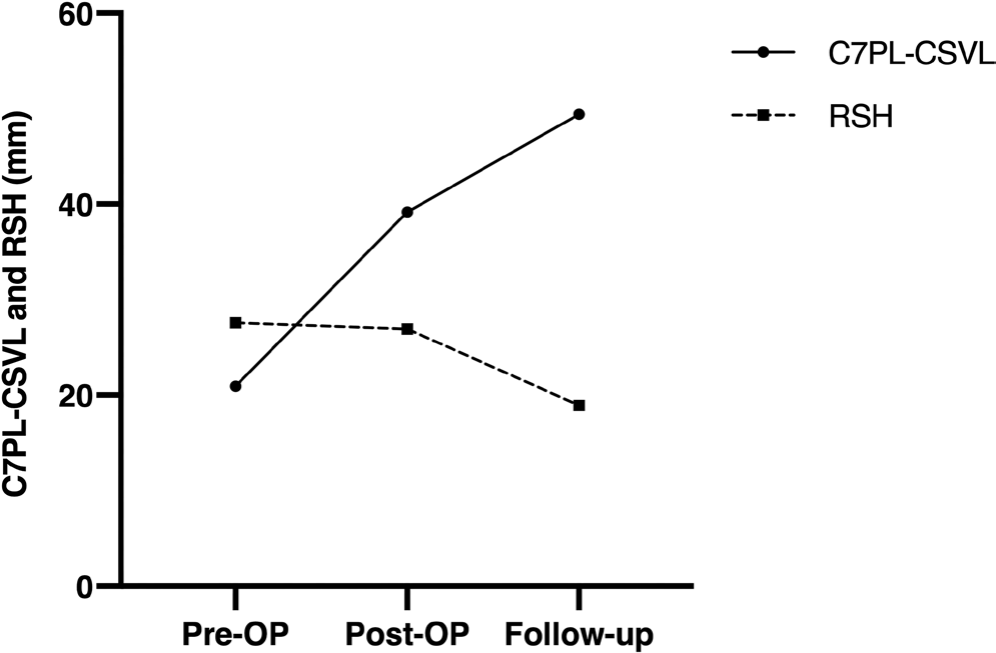
The adverse variation trends of shoulder height difference and C7PL‐CSVL in aggravated group. RSH, radiographic shoulder height.

**FIGURE 7:**
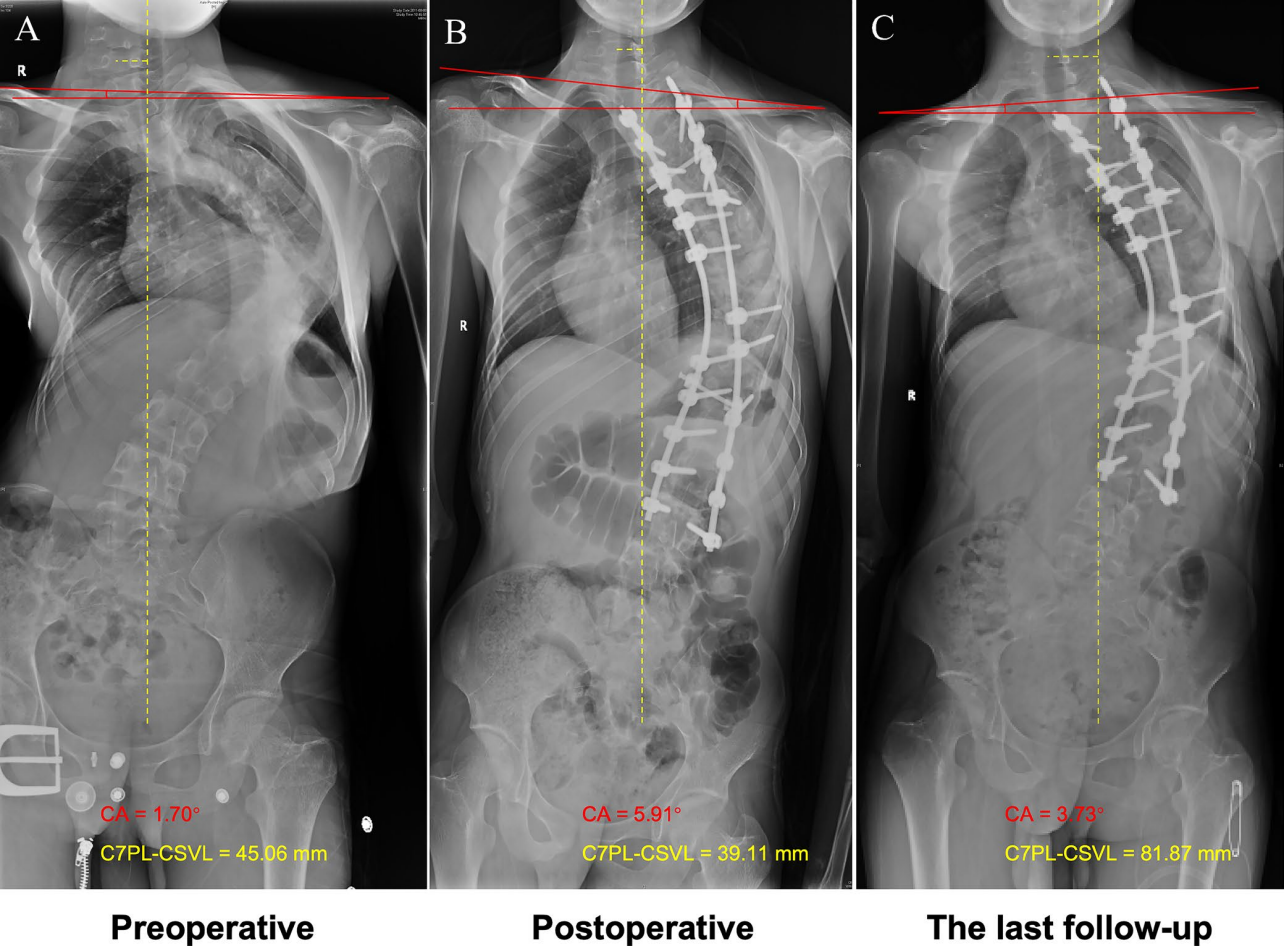
The preoperative, immediately postoperative, and the last follow‐up radiographs of a representative case. Posteroanterior radiographs were obtained in a 13‐year‐old female who was diagnosed with severe and rigid congenital scoliosis and underwent posterior instrumentation. Shoulder imbalance and coronal imbalance were observed preoperatively, and the C7PL‐CSVL was slightly corrected by surgery (45.06 mm to 39.11 mm). However, the patient developed shoulder imbalance (CA = 5.91°) towards the opposite side postoperatively. During the follow‐up, the aggravated coronal imbalance (C7PL‐CSVL = 81.87 mm) was observed, C7PL shifted towards the side of higher shoulder, and the shoulder imbalance was improved (CA = 3.73°).

When we further analyzed the 11 patients whose C7PL‐CSVL aggravated greater than 20 mm relative to the first visit (Aggravated group vs. non‐Aggravated group), this potential compensatory mechanism was reflected more obviously. Between groups, postoperative C7PL‐CSVL had similar values (42.53 ± 24.40 mm vs. 39.17 ± 25.24 mm, *p* = 0.754). However, the postoperative shoulder imbalance in the Aggravated group was more severe (6.13° ± 2.90° vs. 3.70° ± 2.41°, *p* = 0.045, CA; 26.93 ± 15.20 mm vs. 17.58 ± 11.24 mm, *p* = 0.117, RSH), whereas this gap was gradually narrowing during the follow‐up and attained approximately equal at the last measurement (3.46° ± 2.88° vs. 3.09° ± 1.96°, *p* = 0.729, CA; 18.91 ± 11.75 mm vs. 16.93 ± 9.22 mm, *p* = 0.665). By contrast, in terms of C7PL‐CSVL, the difference between groups was gradually increasing during the follow‐up (3.36 ± 10.58 mm to 16.62 ± 8.66 mm), despite not having statistical significance (*p* = 0.069). Thus, the adverse variation trends of shoulder height difference and C7PL‐CSVL confirmed that the aggravating coronal imbalance might be a compensation for shoulder imbalance (Figures 5 and 6). A representative case was available in Figure 7. Therefore, the development of postoperative shoulder imbalance may presage the subsequent onset or aggravation of coronal imbalance during the follow‐up. However, all these hypnoses were based on the observed adverse trends, and the compensatory relationship between shoulder imbalance and coronal imbalance should be verified with caution.

Following previous studies, the pre‐existing coronal imbalance was also identified as a causative factor for coronal imbalance at the last follow‐up [7, 33]. The insufficient restoration of AVT (105.41 ± 32.20 mm to 83.79 ± 34.39 mm) and C7PL‐CSVL (32.59 ± 22.36 mm to 40.85 ± 24.28 mm) led to the residual coronal imbalance in the early postoperative period. The compensatory potential of unfused segments, however, just slightly improved the AVT (7.62 ± 2.69 mm, *p* = 0.02) and C7PL‐CSVL (0.26 ± 3.67 mm, *p* > 0.05) during the follow‐up period. The unfused curve was unable to adequately compensate for the residual postoperative C7PL–CSVL deviation, which consequently resulted in coronal imbalance at the last follow‐up. This finding was consistent with the results of Ishikawa et al. [7], who reported that patients with immediately postoperative coronal imbalance failed to remodel coronal balance by postural reflex or relatively flexible curve during the follow‐up. We believed that the unsatisfactory surgical and rehabilitation effects in complex spinal deformity were further attributed to the severity and rigidity of the curve. Based on the results of risk factor analysis, the predictive equation was developed. The Risk Index of coronal imbalance was 1.922 × postoperative CA + 0.068 × postoperative C7PL‐CSVL, which indicated that the coronal imbalance at the last follow‐up could be predicted by postoperative shoulder height difference and the residual C7PL‐CSVL.

### 5.3 Strategies for Decreasing Risk of Coronal Imbalance

The following strategies may arouse surgeons' attention to prevent the coronal imbalance. In the CIB group, the preoperative shoulder imbalance was also severe (26.16 ± 22.62 mm, RSH; 5.42° ± 5.35°, CA), so we considered that the remained postoperative shoulder imbalance may be a result of undercorrection of the rigid PTC which was inadequate to compensate for the residual shoulder imbalance. Although the correction rate of PTC was similar between groups (*p* > 0.05), more sufficient correction and fusion of the PTC should be emphasized in patients with preoperative shoulder imbalance [34]. In addition, a moderate correction of MTC should also be noted [35]. For the immediately postoperative coronal imbalance, the lower correction rate of TL/LC might be one of the underlying causes (47.9% vs. 63.1%, CIB vs. CB group, *p* = 0.077), which was consistent with the finding of Trobisch et al. [11]. Therefore, we also recommended the sufficient correction of TL/LC for an appropriate ratio of MTC to TL/LC to eliminate this risk factor.

Besides the relationships between PTC, MTC as well as TL/LC, postoperative bracing and balance training were also critical [36]. Self‐position‐adjustment can be used to avoid minor decompensation and shoulder imbalance. We also suggested a closer follow‐up. If deterioration of coronal imbalance was detected, timely careful evaluation and pertinent effective measures had to be taken.

### 5.4 Etiological Homogeneity in Severe and Rigid Scoliosis

A potential concern in the analysis of severe spinal deformity is the heterogeneity of etiology. Our cohort included both severe idiopathic scoliosis and congenital scoliosis (excluding lumbosacral hemivertebrae). Although these conditions differ in embryogenesis, our analysis indicates that they share a convergent biomechanical pathway in the severe and rigid stage that justifies their pooled analysis regarding coronal balance.

First, we observed no statistical difference in the distribution of idiopathic/congenital cases between the CB and CIB groups (idiopathic scoliosis: 55.2% vs. 63.6%; congenital scoliosis: 44.8% vs. 36.4%) (*p* > 0.05). This suggests that in curves exceeding 80° with limited flexibility, the original etiology is not a primary driver of postoperative trunk shift. Instead, geometric parameters such as shoulder balance and residual curve correction predominate. Second, the structural characteristics of “rigidity” minimize the distinction between these etiologies. In severe idiopathic scoliosis, chronic asymmetric loading leads to the degeneration of facet joints and discs on the concave side, often resulting in spontaneous ankylosis or “autofusion”. This biological fusion creates a rigid bony tether functionally indistinguishable from the congenital bars or failures of segmentation. Consequently, the surgical strategy required to mobilize the spine—specifically the use of wide posterior releases, facetectomies, and osteotomies—was uniform across our cohort regardless of diagnosis. Finally, the specific congenital anomalies included in this study (primarily rib synostosis and thoracic hemivertebrae) underwent corrective maneuvers that neutralized their unique deforming potentials. For cases with rib synostosis, thoracoplasty and rib resection were performed to release the chest wall tether, thereby eliminating the restrictive force contributing to the deformity. Similarly, hemivertebra resection physically removes the teratogenic wedge, effectively “normalizing” the spine's biomechanical environment to that of a standard fusion. Because the teratogenic factors were surgically ablated, the postoperative mechanism of maintaining coronal balance in congenital patients became dependent on the same instrumentation principles governing the idiopathic cases.

## 6 Strengths and Limitations

The main strength of this study lies in its specific focus on severe and rigid scoliosis, filling a critical gap in the literature where most risk factor analyses are derived from moderate and flexible cases. Unlike previous studies that focused solely on spinal alignment, this research provides novel insights into the compensatory mechanism between shoulder imbalance and trunk shift in complex spine deformities.

The main limitation of this study is the relatively small sample size. While the predictive model established in this study demonstrates high discriminatory capability (AUC = 0.905) and satisfactory calibration (Hosmer‐Lemeshow test; *p* = 0.844), interpreting these metrics requires a nuanced understanding of the statistical constraints inherent to severe and rigid scoliosis cohorts. The combination of a limited sample size (*n* = 51) and high predictive accuracy necessitates a critical examination of potential overfitting risks. Specifically, the ratio of events to predictor variables (EPV) was approximately 11:1 (22 coronal imbalance events for 2 covariates), exceeding the traditional “rule of 10” threshold for minimizing bias. Nevertheless, the wide 95% confidence interval for the postoperative CA odds ratio (1.659–28.187) reveals a degree of estimation uncertainty inevitable in small cohorts. We attribute this magnitude volatility to the Hauck‐Donner effect—a statistical artifact arising from the variable's strong separation power within a small sample [37]. While the influence of shoulder imbalance on trunk shift remains statistically robust, the precise effect size is subject to shrinkage when applied to external populations. Consequently, in the absence of an external validation cohort, this predictive risk index should be regarded as an internally validated exploratory tool. It effectively highlights the critical link between residual shoulder imbalance and ultimate trunk shift; however, future multi‐center studies with larger sample sizes are required to verify its generalizability and calibrate potential optimism bias.

The use of absolute values for radiographic parameters (CA, RSH, C7PL–CSVL) also represents a limitation. While this approach was used to prevent the mathematical neutralization of left‐ and right‐sided values when calculating cohort means, and to assess the magnitude of imbalance, it inherently obscures the directional nature of the imbalance. Consequently, specific biomechanical coupling patterns—such as whether a right‐sided shoulder elevation consistently predicts a right‐sided trunk shift—could not be fully elucidated in this analysis. Finally, a longer follow‐up period is required to observe the long‐term evolution of coronal balance and to determine whether spontaneous compensation or further decompensation may occur over time.

## 7 Conclusions

In patients with severe and rigid scoliosis, the postoperative coronal imbalance cannot be spontaneously corrected during the follow‐up, and the incidence remained high at the last follow‐up (43.1%). Immediately postoperative C7PL‐CSVL and CA could be used as predictors for the ultimate coronal imbalance. The aggravation of coronal imbalance might represent a possible compensatory response to shoulder imbalance, as suggested by the observed adverse trends. However, this interpretation should be considered hypothetical, since a direct causal relationship could not be verified yet. Our findings highlighted the impact of shoulder imbalance on coronal imbalance and decompensation; surgeons should pay attention to restore an appropriate relationship between PTC, MTC, as well as TL/LC in surgical planning.

## Author Contributions

**Honghao Yang:** data curation, investigation, formal analysis, writing – review and editing, writing – original draft. **Haoshuang Geng:** formal analysis, investigation. **Jie Wang:** data curation. **Jixuan Huang:** data curation, investigation. **Yiqi Zhang:** methodology, formal analysis. **Yunsheng Wang:** methodology, validation. **Yangpu Zhang:** software, validation, formal analysis. **Lijin Zhou:** supervision, resources. **Yong Hai:** conceptualization, methodology, supervision, project administration, resources.

## Funding

This study received fund support from Clinical Research Incubation Project, Beijing Chaoyang Hospital, Capital Medical University (CYFH202218).

## Disclosure

All authors listed meet the authorship criteria according to the latest guidelines of the International Committee of Medical Journal Editors. All authors are in agreement with the manuscript.

## Ethics Statement

This study was approved by the ethics committee and informed consent was obtained from the patients or their parents.

## Conflicts of Interest

The authors declare no conflicts of interest.

## Data Availability

The data that support the findings of this study are available from the corresponding author upon reasonable request.
